# Concurrent Use of Herbal and Orthodox Medicines among Residents of Tamale, Northern Ghana, Who Patronize Hospitals and Herbal Clinics

**DOI:** 10.1155/2018/1289125

**Published:** 2018-03-19

**Authors:** Evans Paul Kwame Ameade, Mohammed Ibrahim, Halimatu-Sadia Ibrahim, Rabiatu Hamisu Habib, Stephen Yao Gbedema

**Affiliations:** ^1^Department of Pharmacology, School of Medicine and Health Sciences, University for Development Studies, Tamale, Ghana; ^2^Department of Nursing, School of Allied Health Sciences, University for Development Studies, Tamale, Ghana; ^3^Department of Herbal Medicine, Faculty of Pharmacy and Pharmaceutical Sciences, Kwame Nkrumah University of Science and Technology, Kumasi, Ghana

## Abstract

Despite the development of more researched and formulated orthodox medicines, herbal medicines continue to be well patronized for persons across the world with some patrons concurrently using both forms, oblivious of the unwanted effects that may occur. Using a multistage sampling procedure, a semistructured questionnaire was used to collect data in April 2016 from 240 informants from three selected hospitals and three herbal clinics in Tamale, a city in northern Ghana. Using Statistical Package for the Social Sciences, binary logistic regression was used to determine sociodemographic predictors of concurrent use of herbal and orthodox medicines. Orthodox medicines were the drug of choice for 54.2% and 49.2% of patrons of hospitals and herbal clinics, respectively. Also, 67.5% of herbal clinic patrons used orthodox medicines, while 25.0% of hospital attendees used herbal medications prior to their visit to the health facilities. Up to 17.9% of respondents concurrently used herbal and orthodox medicines for their prevailing ailment with age, less than 30 years being the only predictor of this habit (*p* = 0.015; 95% CI, 1.183–4.793; cOR = 2.4). All health professionals including those in herbal clinics should therefore be interested in the drug history of their clients.

## 1. Background to the Study

Diseases have afflicted man for ages but humans always make effort to remedy the situation in order to regain a life disturbed by these ailments. The earliest form of healing substances had been herbal medicines, but with the advent of civilization which had led to better scientific understanding of diseases and medications, orthodox medicines have become the main and well recognized products for the management of diseases in modern health systems [1, 2]. According to Mahomoodally (2013), herbal medicines (HM) include herbs, herbal materials, herbal preparations, and finished products that contain parts of plants or other plant materials as active ingredients [3]. Orthodox medicines (OM) or drugs on the other hand are chemically pure substances which when administered into the body produce pharmacological effects which may consequently lead to alleviation of the disease or help in the diagnosis or prevention of the disorder [4]. Many current orthodox drugs have their origin from herbal medicines, but the main difference between the two is that the herbal drugs contain a large number of compounds, rather than a single pharmacologically active substance; hence components of both herbal and orthodox medicines may act on one another to moderate, oppose, or enhance an effect [5, 6]. It would have been expected that orthodox medicine should be an overwhelming favoured choice of treatment of diseases since it is a more refined and scientifically studied remedy. Herbal medicines however also continue to be well patronized in both developing and developed countries of the world. According to the World Health Organization (2002), despite the introduction of orthodox medicine by the Europeans who colonized Africa, up to 80% of Africans still use traditional medicines, especially herbal medicine for their primary healthcare needs [7]. Patronage of herbal products has also seen an increase even in developed countries such that approximately 20% of people in the United States of America use herbal products for various health reasons [8]. Despite the high patronage of herbal medicines all over the world, patients do not inform their doctors about their use of orthodox medicine concurrently with these herbal products and also most doctors do not also enquire from the patients about the use of herbal products during consultation [9–12]. Concurrent use of herbal and orthodox medicines according to Neustadt (2006) causes interactions between these two forms of medicines which can lead to undesirable pharmacokinetic and pharmacodynamic effects [13]. For example, when a herbal preparation containing St. John's Wort* (Hypericum perforatum)* is administered together with digoxin, there is always a significant decrease in maximum serum concentration of digoxin and hence its efficacy because the plant product increases digoxin metabolism. Again, the anticoagulant effect of warfarin is enhanced when taken together with ginkgo* (Ginkgo biloba)*, thereby increasing the possibility of excessive bleeding [1, 13]. Issues of adverse effects and drug-herb interactions should be of important public health concerns because of their overall effect on human health and safety. In Ghana, efforts are being made to integrate herbal medicines into the orthodox health facilities to provide alternative system for individuals who for some reasons would want to access alternative medicines for their healthcare needs. The operationalization of the Traditional Medicine Practice Council (TMPC) in 2010 following the passing of the Traditional medicine practice Act, Act 575 in 2000, and the subsequent commencement of training of medical herbalists by Kwame Nkrumah University of Science and Technology in Ghana in 2001 had accelerated the integration of herbal medicine into the Ghanaian orthodox health system [14]. Beside the herbal clinics in some teaching and regional hospitals, there are privately owned herbal clinics that provide alternative healthcare systems to Ghanaians. These herbal clinics give their clients herbal products. Just as persons who attend orthodox hospitals are given orthodox drugs which could be concurrently taken with herbal medicines, those who patronize herbal clinics would possibly be taking these herbal products with some orthodox medicines as well. Most studies on the concomitant use of herbal and orthodox medicines were carried out in hospitals or communities where orthodox medicines are prescribed for patient or individuals who self-medicate with them [15–18]. There is currently paucity of information on whether persons who visit the herbal clinics also concurrently use orthodox medicines; hence this study assessed the level of concurrent usage of herbal and orthodox medicine among patients at hospitals and herbal clinics in Tamale and ascertained the predictors of this habit among the respondents.

## 2. Method

### 2.1. Study Design and Setting

A cross-sectional survey to collect data on the level of concurrent usage of herbal medicines and orthodox medicines was undertaken in three public hospitals and the three most patronized herbal clinics in Tamale, a city in northern Ghana which according to the Ghana Statistical Service (GSS) 2010 population and housing census has a total population of 223,252 [19]. The hospitals surveyed were the Tamale Teaching Hospital, Tamale West Hospital, and Tamale Central hospital, while the herbal clinics were Alive Legacy Herbal Clinic, Ameen Scientific Herbal clinic, and Unique Naturalist Herbal Clinic.

### 2.2. Study Tool

The study was conducted in April 2016 using a semi-structured questionnaire. The questionnaire was pretested among 20 clients, which ensured the correction of ambiguous and inconsistent questions before it was administered for the actual data collection. The authors reviewed the questionnaire to ensure face validity of the data collecting tool. Though most of the final questionnaires were self-administered, a few were administered employing the face-to-face interview questionnaire administration method with respondents and retrieved immediately after completing the questions. The face-to-face interview questionnaire was used for respondents who were not literate enough to complete the questionnaire on their own.

### 2.3. Study Sample Size Determination

Cochran's (1977) formula was used to estimate a desirable sample size. The sample size was calculated as follows: *n* = *z*^2^(1 − *p*)*p*/*d*^2^, where *n* is the sample size, *z* is the standard normal distribution taken as 1.96, *p* is the estimated prevalence rate of herbal users being 80% (0.80), and *d* is the margin of error equal to 0.05. *n* = 1.96^2^(1 − 0.8)0.8/0.05^2^; therefore, the minimum sample size for this study was 245.9 = 246.

### 2.4. Sampling Procedure

The sampling method that was employed in this study was the multistage sampling procedure. The first stage consisted of two clusters, named A and B. Cluster A represented those who attend herbal clinics and cluster B represented those who attend hospitals. Respondents in B were outpatients and inpatients, while respondents in A were all outpatients, since A study sites do not have inpatients services. In the second stage, a convenient sampling method was applied with the numbers for each facility apportioned based on the daily attendance records. The sample was divided among clusters A and B in a ratio of 1 : 1.

### 2.5. Statistical Analysis

The data collected was grouped for editing and keyed into Microsoft Excel (2013 version). Data was analyzed using Statistical Package for the Social Sciences (SPSS), version 20 (SPSS Inc, IBM, Chicago, IL, USA). Association between variables was determined using binary logistic regression. Statistical significance was assumed at *p* < 0.05 at a confidence interval of 95%.

## 3. Results

### 3.1. Sociodemographic Profile of Respondents

Table 1 shows the sociodemographic characteristics of respondents in this study. The majority were as follows: females, 127 (52.9%), followers of the Islamic religion, 154 (64.2%), those who grew up in rural areas of Ghana, 122 (50.8%), and persons in the low-income bracket, 132 (55.0%), as indicated by their living accommodation being single rooms in a compound house. Most respondents were between 21 and 30 years old, 104 (43.3%); currently married, 111 (46.3%); and self-employed, 89 (37.1%), and had tertiary level education, 73 (30.4%).

### 3.2. Reasons for Concurrent Usage of Herbal and Orthodox Medicines

The level of concurrent usage of herbal medicines and orthodox medicines and the reasons for this practice are as shown in Table 2. For hospital participants, prior to the visit, the minority, 30 (25.0%), used herbal medicines for their current sickness, while the majority of herbal clinic patrons, 81 (67.5%), used orthodox medicine before visiting the herbal clinic. For all the participants, only 43 (17.9%) were concurrently using both herbal and orthodox medicines. The orthodox doctors or herbal practitioners did not bother to ask the majority of these participants, 27 (62.8%), if they were concurrently using both orthodox and herbal medicines. For those concurrently using both orthodox and herbal medicines, the majority, 25 (58.1%), do not intend to stop that practice, with a further 11 (25.8) not sure of stopping or continuing. The top two reasons for concurrent use of herbal and orthodox medicines were the following: both medicine types work together for the management of the condition, 10 (25.0%), and the combination of both forms of medicines was more effective in treating the prevailing disease condition, 9 (23.1%).

### 3.3. Reasons for Participants' First Choice of Medicines When Sick

The majority of hospital attendees, 65 (54.2%), and 59 of the herbal clinic attendees (49.2%) would first opt for orthodox medications when sick. The top three reasons why hospital attendees would first opt for orthodox medicines were the following: the medicines being more effective (27.9%), better studied, and approved (22.1%) and clearer dosage (14.7%). For herbal clinic attendees, their top three reasons were as follows: easy accessibility of orthodox medicines (18.5%), faster curing (15.4%), and clearer dosage (13.8%). The minority of both hospital and herbal clinic attendees, 42 (35.0%), would opt for herbal medicines as the first choice of medication when unwell. The top three reasons why patrons of hospitals would first opt for herbal medicines when sick were as follows: they were more effective (28.8%), they cure faster (15.2%), and they bring complete cure (10.6%). For the herbal clinic attendees, herbal medicines being more effective (22.1%), having no or lesser side effects (13.2%), curing completely (13.2%), and curing faster (13.2%) were their main reasons for choosing herbal medicines over orthodox medicines.  Table 3 shows the type of medications respondents would first opt for whenever they fall sick and the reasons for their choices.

Nine variables describing the sociodemographic characteristics of the respondents, namely, sex, age, religion, location of stay when growing up, marital status, educational level, employment status, income level, and the type of health facility, whether it was an orthodox hospital or a herbal clinic, were analyzed against concurrent use of herbal and orthodox medicines using binary logistic regression (Table 4). Age of the respondents was the only factor that was significantly associated with the concurrent use of herbs and orthodox medicines (*p* = 0.015) with persons less than 30 years old almost 2.4 times more likely to co-use the two types of medicines (OR, 2.381; 95% CI, 1.183–4.793). Although more persons who sought healthcare services from hospitals concurrently used both herbal and orthodox medicines more than those at herbal clinics (20.5% versus 16.9%), the difference was not statistically significant.

## 4. Discussion

Technological advancement had led to better understanding of diseases which had led to development of pure chemicals which have been used to formulate orthodox medicines mostly used in modern health facilities across the world. It would have been expected that crude natural products or alternative medicines would see a decline in usage, but studies have shown a rather increased usage across the world [20–23]. Despite the increasing patronage of herbal products across the world, this study found that the majority was of both hospital clients (54.2%), while most (49.2%) of herbal clinic attendees opted for orthodox medicines as their first choice of medication when unwell. A study in communities in Orlu Local Government Area in the Imo state and Abuja both in Nigeria found that 70.4% and 86.3%, respectively, opted first for orthodox medicines [17, 18]. With increased availability of orthodox medicines and the increasing level of interest in herbal medicines, there is a tendency for both forms of drugs, being concurrently used or one form is used prior to the use of the other one. This study found that two-thirds of patients attending herbal clinics used orthodox medicines prior to their visit to the facilities, while only a quarter of hospital attendees used herbal medicines preceding their visits. What could have accounted for the high level of orthodox medication among the herbal clinic attendees and the lower level of herbal product usage by the hospital attendees is that orthodox medicines are more accessible in a city as Tamale, where over-the-counter medicine sellers and pharmacies are within walking distance of homes unlike the herbal shops. Accessibility to orthodox medicines was the most cited reason by herbal clinic attendees for opting for this form of therapy as first choice when sick. Although it is unclear if the patrons of the herbal clinics who had used orthodox medicines prior to their visit got them based on a physician's prescription or self-medication and even whether they administered the drugs using the right dosage regimen, their decision to seek healthcare at herbal clinics could possibly be due to their lack of satisfaction with the orthodox medications. This assertion is supported by the results of this study which found that both hospital and herbal clinic attendees had their topmost reason for opting for herbal medicines being that herbal medicines are more effective than orthodox medicines. Lack of satisfaction with orthodox medicines had also been found to be a major reason for people opting for herbal medicines in one study in the USA [20]. This study recorded about a fifth (17.9%) of respondents concurrently using both herbal and orthodox medicines which is lower than results from countries such as Nigeria, Kenya, and Norway where between 25% and 69.4% concurrently used both herbal and orthodox medicines [15–18, 24]. The possible reason for the lower rate in this study could be because the responses given by the participants were in relation to the concurrent use of both forms of medication for the ailment for which they had gone to seek healthcare but most other studies questioned respondents if they had ever concurrently used herbal and orthodox medicines. For more than half of respondents in this study who concurrently use HM and OM not intending to stop the practice and almost two-thirds of their physicians or herbal practitioners not asking them of their concurrent use of these two types of medicines gives some cause to worry. Residents in Tamale who seek healthcare services either in the hospitals or in the herbal clinics are therefore invariably exposed to greater chances of suffering the negative effects of drug-herb interactions. The fact that most respondents also stated that concurrent use of herbal and orthodox medicines is better and a more effective way of treating their conditions brings to the fore the need for greater education to lessen the consequences of drug-herb interactions. Several studies in Nigeria and Norway found an association between concurrent use of herbal and orthodox medicines and several sociodemographic characteristics including age, sex, level of education, and income level [15–17]. This study however found age of respondents as the only factor associated with the concurrent use of herbal and orthodox medicines with individuals younger than 30 almost 2.4 times more likely to combine the two forms of drugs than those older than 30 years. This is contrary to studies by Djuv et al., (2013), Duru et al., (2016), and Githinji (2016) who found that concurrent usage of herbal and orthodox medicines increases with age [16, 17, 24]. The reason for the differences based on age could be that persons younger than 30 years in this study were more reluctant to seek healthcare services when sick, would self-medicate with all forms of drugs, and would only visit healthcare facilities when the situations become unbearable. A study in Brazil also showed that persons younger than 30 years significantly self-medicate [25]. The concurrent use of herbal and orthodox medicines by persons who attend hospitals and other orthodox health facilities is well documented [15–18] but not much can be found about the situation for those who attend herbal clinics. A study in the Githunguri Division of Kiambu Count in Kenya among patrons of herbal clinics also showed a high level (42.5%) of orthodox medicines, a number higher than the 20.5% reported in this study [24]. The variation in the result (42.5% versus 20.5%) could be attributed to the difference in location since the Githunguri division is a rural area, while Tamale is a city. Access to orthodox medicines is more controlled in cities than rural areas of Ghana where drug peddlers move around to illegally dispense orthodox medicines; hence rural dwellers easily acquire these medicines which they sometimes concurrently take with the herbal preparations [26]. Millions of people across the world would continue to depend on herbal medicines for the management of their health conditions [27] so the patronage of the increasing number of herbal clinics in Ghana would continue. With this study showing that the place of seeking healthcare services did not have any association with concurrent use of HM and OM, it is important that besides orthodox healthcare practitioners, herbal medicine practitioners should also be sensitized to be interested in the drug history of their clientele and then educate them on the dangers of taking these forms of medications together. Although, this is possibly the first study in Ghana which assessed the concurrent use of OM and HM among patrons of orthodox medical practice centres and herbal clinics, several limitations could affect the adequacy and generalized interpretation of the results of this study. Firstly, the use of self-administered questionnaire in some cases rather than interviews for all makes verification of the answers provided by the respondents difficult. Again, some respondents could neither read nor write which requires a translation into a language the respondents understand. Errors of misinterpretation and recording of responses could also have affected the results. However, the results from this study should serve as a starting point for a nationwide study on the use of concurrent use of HM and OM among patrons of herbal clinics and how significant the level of this habit is when compared to patrons of orthodox medical systems.

## 5. Conclusion

Prevalence of the concurrent use of herbal and orthodox medicines in this study is low but when sick, almost half of respondents would opt for orthodox medicines. All the sociodemographic characteristics of respondents except the age had no association with respondents' concurrent usage of herbal and orthodox medicines. Patients less than 30 years were almost 2.4 times more likely to concurrently use both herbal and orthodox medicines than those above 30 years but the place of seeking healthcare system, whether orthodox medical systems or herbal clinics, had no association with this habit. Belief that both herbal medicines and orthodox medicines work together to manage an ailment is the main reason for those who use both forms of medications.

## Figures and Tables

**Table 1:** Sociodemographic characteristics of respondents.

Variable	Subgroup	Frequency	Percentage
Sex	Female	127	52.9
	Male	111	46.3

Religion	Christianity	80	33.3
	Islam	154	64.2
	Traditionalist	3	1.3

Age	<21	14	5.8
	21–30	104	43.3
	31–40	42	17.5
	41–50	33	13.8
	51–60	21	8.8
	>60	25	10.4

Marital status	Single	91	37.9
	Currently married	111	46.3
	Ever married	30	12.5

Educational status	None	74	30.8
	Basic	25	10.4
	Secondary/technical	52	21.7
	Tertiary	73	30.4

Location of growing up	Rural	117	48.8
	Urban	122	50.8

Employment status	Unemployed	30	12.5
	Students	45	18.8
	Housewife	24	10.0
	Private sector	20	8.3
	Self-employed	89	37.1
	Public sector	28	11.7
	Retiree	2	0.8

Type of accommodation currently occupied^*∗*^	Single room in compound house	132	55.0
	Chamber and hall in compound house	65	27.1
	Self-contained apartment	36	15.0
	Mansion	4	1.7

^*∗*^Used as proxy to measure income level. Low income earners occupied single rooms and chamber and hall apartments. Those in self-contained apartments and mansions were middle to high income earners.

**Table 2:** Concurrent use of both herbal and orthodox medicines.

Variable	Subgroup	Frequency	Percentage
Have you ever used herbal medicine (HM) for current sickness before coming to the hospital? (*n* = 120)	Yes	30	25.0
	No	86	71.7

Have you ever used orthodox medicine (OM) for current sickness before coming to the herbal clinic? (*n* = 120)	Yes	81	67.5
	No	37	30.8

Are you concurrently using HM and OM for current ailment?	Yes	43	17.9
	No	187	77.9

Did orthodox doctor or herbal doctor ask you of current usage of HM and OM? (*n* = 43)	Yes	12	27.9
	No	27	62.8

Do you intend stopping concurrent use of HM and OM?	Yes	7	16.3
	No	25	58.1
	Not sure	11	25.6

What are the reasons for concurrent use of HM and OM? (*n* = 39)	Both work together to manage my condition	10	25.6
	Make me more comfortable about managing my condition	2	5.1
	More effective in treating diseases	9	23.1
	Others	4	10.3
	Quicken recovery	7	17.9
	Treatment cost is lower	7	17.9

**Table 3:** Participants' first choice of medicines when sick and the reasons for the choices.

Variable	Subgroups	Hospital attendee		Herbal clinic attendee	
		Frequency	Percentage	Frequency	Percentage
First medication choice when sick	Orthodox	65	54.2	59	49.2
	Herbal	42	35.0	42	35.0
	Uncertain	13	10.8	19	15.8

Reasons for choosing orthodox medicine first (*n* Hosp. = 68; *n* herb = 65)	Accessible	5	7.4	12	18.5
	Clear dosage	10	14.7	9	13.8
	Well studied and approved	15	22.1	8	12.3
	More effective	19	27.9	4	6.2
	Condition requires medical care	4	5.9	NA	NA
	Safer	7	10.3	6	9.2
	More confidence in OM	3	4.4	NA	NA
	Recommended	2	2.9	NA	NA
	Others	3	4.4	5	7.7
	Cures faster	NA	NA	10	15.4
	Easy and convenient	NA	NA	3	4.6
	Personal preference	NA	NA	2	3.1
	Prepared hygienically	NA	NA	4	6.2
	Variety of forms	NA	NA	2	3.1

Reasons for choosing herbal medicine first (*n* hosp. = 66; *n* herb = 68)	Accessibility	4	6.1	3	4.4
	Affordability	6	9.1	7	10.3
	Heritage	6	9.1	NA	NA
	More effective	19	28.8	15	22.1
	No or lesser side effects	6	9.1	9	13.2
	OM is unable to manage all diseases	2	3.0		
	Cures completely	7	10.6	9	13.2
	Cures faster	10	15.2	9	13.2
	Natural	2	3.0	5	7.4
	Less complicated	NA	NA	2	2.9
	Personally like herbal medicines	NA	NA	4	5.9
	Others	4	6.1	5	7.4

*NB*. *n* Herb = respondents from the herbal clinics, *n* hosp. = respondents from the hospitals, and NA = not applicable.

**Table 4:** Sociodemographic predictors of concurrent usage of orthodox and herbal medicines.

Variable	Subgroup	Have you ever used herbal and orthodox medicines concurrently?			% of concurrent users of HM and OM	*p* value	Crude odd ratio (95% CI)
		Yes	No	Total number			
Sex	Female	23	98	121	19.0	0.925	1.033 (0.531–2.008)
	Male^Ref^	20	88	108	18.5		

Age	<30	29	87	116	25.0	0.015^*∗*^	2.381 (1.183–4.793)
	>30^Ref^	14	100	114	12.3		

Religion	Christianity	13	67	80	16.3	0.46	0.763 (0.373–1.563)
	Islam^Ref^	29	116	145	20.0		

Location	Urban^Ref^	21	96	117	17.9	0.768	1.105 (0.569–2.145)
	Rural	22	91	113	19.5		

Marital status	Single^Ref^	20	70	90	22.2		
	Currently married	20	84	104	19.2	0.082	0.259 (0.057–1.185)
	Ever been married	2	27	29	6.9	0.608	0.833 (0.415–1.672)

Educational status	No formal education	8	61	69	11.6	0.390	0.656 (0250–1.718)
	Basic	3	21	24	12.5	0.628	0.714 (0.183–2.781)
	Secondary	12	38	50	24.0	0.318	1.579 (0.644–3.374)
	Tertiary^Ref^	12	60	72	16.7		

Employment status^a^	Unemployed^Ref^	23	76	99	23.2		
	Self-employed	15	69	84	17.9	0.373	0.718 (0.347–1487)
	Employed	5	41	46	10.9	0.086	0.403 (0.143–1.139)

Income status	Low income^Ref^	35	153	185	18.6		
	Middle to high income	8	32	40	20.0	0.839	1.093 (0.464–2.576)

Place of seeking health	Hospital^Ref^	23	89	112	20.5		
	Herbal clinic	20	98	118	16.9	0.486	0.790 (0.406–1.535)

Ref: reference variable, ^*∗*^statistically significant, and ^a^Unemployed, students, housewives, and retirees were grouped as unemployed, while private and public sector workers were considered as employed.
